# Functional and Structure Prediction of Hypothetical Proteins From *Listeria aquatica* FSL S10‐1188: Bioinformatics Approaches

**DOI:** 10.1155/bmri/1235637

**Published:** 2025-12-28

**Authors:** Mutaz Mohammed Abdallah, Ruaa Abdalla Ibrahim Suliman, Yousra Tagelsir Ahmed, Mawada Yahia

**Affiliations:** ^1^ Department of Microbiology, College of Life Sciences, Northeast Forestry University, Harbin, Heilongjiang, China, nefu.edu.cn; ^2^ School of Pharmaceutical Science and Technology, Tianjin University, Tianjin, China, tju.edu.cn; ^3^ Microbiology Department, Faculty of Medical Laboratory Science, University of Alzaiem Alazhari, Khartoum, Sudan; ^4^ Department of Basic Sciences, Faculty of Dental Medicine and Surgery, National University, Khartoum, Sudan, inu.ac.kr

**Keywords:** AlphaFold3, bioinformatics, GyrI-like detoxification, hypothetical protein, Imm48 immunity, *Listeria aquatica*, molecular docking, protein–protein interactions

## Abstract

**Background:**

*Listeria aquatica* FSL S10‐1188 is a freshwater bacterium characterized by distinctive metabolic properties and a genome enriched in hypothetical proteins (HPs). Although it is not considered pathogenic, its genomic features and aquatic habitat raise concerns about potential involvement in horizontal antimicrobial resistance (AMR) gene transfer.

**Methods:**

The genome of *L. aquatica* FSL S10‐1188 (GenBank Accession: CP011539.1) contains 2789 predicted proteins that were initially screened. From these, 919 HPs were prioritized based on sequence length (> 50 amino acids), physicochemical stability assessed using ProtParam, and consistent subcellular localization predictions from CELLO, PSORTb, and PSLpred. Among these, two stable cytoplasmic, HPs EUJ18943.1 and EUJ18676.1, were selected for comprehensive characterization. Functional insights were explored through BLASTp, secondary structure prediction, and 3D structural modeling using AlphaFold3. Model quality was validated using multiple tools including SAVES v6.0 and QMEANDisCo. Virulence potential was predicted via VICMpred, VirulentPred, and DeepVF. Putative ligand‐binding sites were identified using PrankWeb, and molecular docking analyses were conducted using ProBiS for ligand prediction and AutoDock to evaluate ligand‐binding affinity.

**Results:**

EUJ18943.1 (136 aa) and EUJ18676.1 (206 aa) are stable, hydrophilic proteins with acidic isoelectric points. BLASTp identified EUJ18943.1 as homologous to Imm48 immunity proteins and EUJ18676.1 as a GyrI‐like detoxification protein. Both showed alpha‐helix‐rich secondary structures, with high‐confidence AlphaFold3 models (pTM scores: 0.87 and 0.94). Structural validation confirmed the model quality. Virulence prediction tools classified both as potential virulence factors. Active sites were predicted by PrankWeb and ProBiS, identifying kanamycin A (score: 2.32) and streptomycin (score: 2.10) as top ligands. AutoDock v4.5.6 revealed strong binding affinities (*Δ* *G* = −7.17 and − 4.30 kcal/mol, respectively).

**Conclusion:**

This study provides the first in silico structural and functional characterization of two HPs from *L. aquatica* FSL S10‐1188, suggesting roles in stress response and virulence. These findings highlight their potential in AMR gene transfer and demonstrate the importance of computational annotation for guiding future experimental validation.

## 1. Introduction

*Listeria aquatica* is a Gram‐positive, facultatively anaerobic, nonmotile, nonspore‐forming rod‐shaped bacterium with distinct characteristics that set it apart from other *Listeria* species [1, 2]. One of its unique attributes is its ability to ferment maltose, making it the only member of the *Listeria* genus with this capability [1]. Additionally, it is the only nonmotile *Listeria* species capable of fermenting D‐tagatose. Unlike many *Listeria* species, *L. aquatica* cannot grow at low temperatures, which is atypical for the genus [3]. The physiological variations indicate niche‐specific adaptations, making *L. aquatica* a suitable subject for comparative genomic and functional research.

*L*. *aquatica* has lower virulence than *Listeria monocytogenes* and lacks antibiotic‐resistant genes. Although its clinical importance is minimal, its occurrence in marine ecosystems may signify possible pollution sources posing dangers to human health [4]. The sequencing of *L. aquatica*′s genome has provided valuable insights for comparative analysis*. L. aquatica* FSL S10‐1188 is a strain belonging to the genus *Listeria*. Notably, it is the sole nonmotile *Listeria* species that can metabolize D‐tagatose through fermentation [5]. *L. aquatica* FSL S10‐1188 was initially isolated from running water, indicating its natural habitat is freshwater environments [1, 6]. This aquatic origin is reflected in its species name, “aquatica.” Recent whole‐genome sequencing (WGS) of FSL S10‐1188 has revealed a genome size of approximately 3.1 Mb, comprising a high GC content typical of *Listeria* species. The sequencing data has allowed researchers to identify multiple coding sequences related to stress responses and nutrient acquisition, and numerous genes annotated as HPs [7].

Hypothetical proteins are predicted protein‐coding genes with unknown functions [8]. The genome of *L. aquatica* FSL S10‐1188 contains thousands of predicted protein‐coding genes, many annotated as HPs [1, 9]. These proteins lack experimental evidence or functional characterization and are identified through computational gene prediction methods but have not been verified experimentally.

Similar approaches could be applied to *L. aquatica* HPs. Bioinformatics tools and techniques to annotate HPs in other bacteria, such as those described for *L. monocytogenes*, could be used for *L. aquatica* HPs [10]. These methods include analyzing physicochemical properties, predicting subcellular localization, and determining active sites. Some HPs in *L. aquatica* may be part of bacteriocin gene clusters or other defense systems, as similar discoveries have been made in related species using advanced bioinformatics algorithms [11].

When experimental evidence is lacking, molecular docking is an essential computational method for predicting ligand–protein interactions. It offers important insights into the binding affinity, specificity, and possible biological roles of uncharacterized proteins [12, 13]. Based on structural integrity and expected virulence potential, we used a multitiered bioinformatics pipeline to identify and model two high‐confidence HPs (EUJ18943.1 and EUJ18676.1) from *L. aquatica* FSL S10‐1188. The ProBiS web server was used to predict ligand‐binding sites in order to clarify their functional roles, and the results showed that streptomycin and kanamycin A were the top‐ranked ligands.

Comprehending the roles of HPs can enhance our understanding of bacterial physiology and facilitate the identification of novel therapeutic targets or diagnostic indicators. This work underscores that bioinformatics predictions necessitate additional experimental validation. Upon completion of this validation, prospective therapeutic applications for targeting these proteins will be elucidated.

## 2. Materials and Methods

### 2.1. Sequence Retrieval and Hypothetical Protein Selection

**Figure 1:**
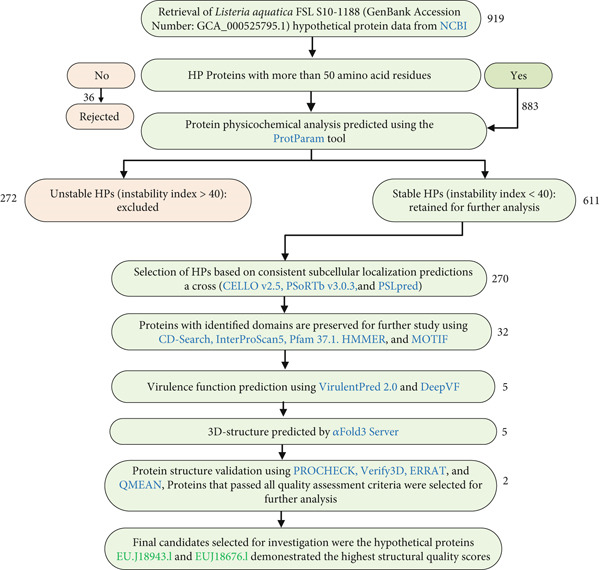
Screening and selection process of hypothetical proteins (EUJ18943.1 and EUJ18676.1). The figure illustrates the stepwise workflow for filtering and identifying the potential HPs from the initial dataset.

The genome of *L. aquatica* FSL S10‐1188 (GenBank Accession Number: GCA_000525795.1), encoding 2789 genes, was retrieved from the National Center for Biotechnology Information (NCBI) [14]. Among these genes, 919 were identified as HPs. The sequences of these HPs were retrieved in FASTA format for further analysis. To enhance the reliability of functional predictions, proteins shorter than 50 amino acids were excluded, as such short sequences are generally incapable of forming stable three‐dimensional structures. After this filtration, 883 HPs with sequences longer than 50 residues were retained for detailed examination (Supporting Information 1: Data S1). The physicochemical properties of these selected HPs were assessed using the ProtParam tool. Based on the analysis, approximately 272 HPs were excluded due to predicted instability. The rest of the 611 stable HPs underwent subcellular localization investigation with three computational tools: CELLO v2.5, PSORTb v3.0.3, and PSLpred. To ensure reliability, only those proteins that exhibited consistent localization predictions across all three tools were retained for further analysis. As a result, 314 HPs showing conflicting or incongruent localization predictions were excluded from subsequent steps (Supporting Information 2: Data S2). The remaining proteins with consistent localization predictions were further analyzed for functional domains using the NCBI Conserved Domain Search (CD‐Search), InterProScan5, Pfam 37.1, HMMER, and MOTIF. This analysis excluded an additional 238 HPs due to a lack of significant domain similarities (Supporting Information 3: Data S3). The remaining proteins were then subjected to virulence prediction using three distinct tools: VICMpred, VirulentPred 2.0, and DeepVF. Five HPs demonstrated consistent virulence profiles across all three tools and were selected for structural prediction using the AlphaFold server. The 3D structures of these five proteins were predicted, and the corresponding PDB files were subjected to quality assessment using PROCHECK, Verify3D, ERRAT, and QMEAN. Two of the five proteins passed all four quality assessments, demonstrating optimal structural stability and accuracy. These two proteins, EUJ18943.1 (136 amino acids) and EUJ18676.1 (206 amino acids), were identified as the top candidates for experimental validation due to their superior structural and functional characteristics (Figure 1 and Table 1) containing all the tools and servers used in this investigation.

**Table 1:** Bioinformatics server and database used in the study.

**No.**	**Server/database**	**Link**	**References**
1	NCBI	https://www.ncbi.nlm.nih.gov/	[14]
2	ProtParam	http://web.expasy.org/protparam/	[15]
3	CELLO v.2.5	http://cello.life.nctu.edu.tw/	[16]
4	PSORTb v.3.0.3	https://www.psort.org/psortb/	[17]
5	PSLpred	https://webs.iiitd.edu.in/raghava/pslpred/submit.html	[18]
6	CDD	https://www.ncbi.nlm.nih.gov/cdd/	[19]
7	Pfam 37.0	http://pfam.xfam.org/	[20]
8	InterProScan	https://www.ebi.ac.uk/interpro/search/sequence/	[21]
9	Motif	https://www.genome.jp/tools/motif/	[22]
10	SOPMA	https://npsa.lyon.inserm.fr/cgi-bin/npsa_automat.pl?page=/NPSA/npsa_sopma.html	[23]
11	PSIPRED	http://bioinf.cs.ucl.ac.uk/psipred/	[24]
12	SWISS‐MODEL	https://swissmodel.expasy.org/	[25]
13	ERRAT	https://saves.mbi.ucla.edu/	[26]
14	VARIFY 3D	https://saves.mbi.ucla.edu/	[27]
15	PROCHECK	https://saves.mbi.ucla.edu/	[28]
16	QMEAN	https://swissmodel.expasy.org/qmean/	[22]
17	PrankWeb	https://prankweb.cz/	[29]
18	VICMpred	https://webs.iiitd.edu.in/raghava/vicmpred/index.html	[30]
19	VirulentPred 2.0	https://bioinfo.icgeb.res.in/virulent2/	[31]
20	DeepVF	https://deepvf.erc.monash.edu/	[32]
21	STRING v.12.0	https://string-db.org/	[33]
22	AlphaFold3	https://alphafoldserver.com/	[34]
23	HMMER	http://hmmer.org/	[35]
24	CLC Sequence Viewer v.8	https://clc-sequence-viewer.software.informer.com/8.0/	—
25	TOPCONS	https://topcons.cbr.su.se/	[36]
26	UCSF ChimeraX	https://www.rbvi.ucsf.edu/chimerax/	[37]
27	ProBiS	http://probis.cmm.ki.si/	[38]
28	PubChem	https://pubchem.ncbi.nlm.nih.gov/	[39]
29	AutoDock 4.5.6	https://autodock.scripps.edu/download-autodock4/	[40]
30	PyMOL	https://www.pymol.org/	[41]
31	LigPlot v.2.3	https://www.ebi.ac.uk/thornton-srv/software/LigPlus/	[42]

### 2.2. Physiochemical and Subcellular Localization Analysis

The ProtParam tool from ExPASy was used to analyze the chemical and physical properties of the selected HPs, including molecular weight, aliphatic index, extinction coefficient, formula, estimated half‐life, theoretical isoelectric point (pI), and grand average of hydropathicity (GRAVY) [15]. Subcellular localization predictions were generated using CELLO [16]. Results were compared with predictions from PSORTb v3.0.3 [17] and PSLpred [18]. Conversely, tools like PSORTb and PSLpred determine the subcellular localization of prokaryotic proteins by evaluating factors such as amino acid composition, dipeptide patterns, physicochemical characteristics, and evolutionary insights obtained through PSI‐BLAST. Both CELLO and PSLpred employ support vector machines (SVMs) for classifying proteins based on these attributes and integrate several computational approaches to improve the precision of localization predictions.

### 2.3. Identification of Homologs and Phylogenetic Analysis

A BLASTp search against the NCBI nonredundant (nr) database was performed to identify homologs of the HPs EUJ18943.1 and EUJ18676.1 [43] using default parameters and local sequence alignment. High‐confidence hits (e‐value < 0.001, query coverage > 70%) were selected for further analysis. Multiple sequence alignment (MSA) was conducted using CLC Sequence Viewer (https://clc-sequence-viewer.software.informer.com/8.0/), and a phylogenetic tree was constructed to assess evolutionary relationships. The tree was visualized and annotated in the same software for clarity.

### 2.4. Functional Annotation of Hypothetical Proteins

Functional annotation was performed using a range of publicly accessible tools and databases to elucidate the functions of the HPs. Conserved domains were identified using resources such as the Conserved Domain Database (CDD) [19], Pfam v.37.1 [20], InterProScan [21], and HMMER web server [35]. CDD was used to identify conserved domains via BLAST‐based alignments and position‐specific scoring matrices (PSSMs). Pfam v37.1 applied hidden Markov models (HMMs) to classify proteins into families and clans. InterProScan5 integrated results from multiple databases (e.g., Pfam, PROSITE, and SMART) using HMMs and PSSMs, providing comprehensive domain and motif annotations along with Gene Ontology (GO) terms. GenomeNet′s Motif Search [22] identified conserved motifs through HMMs and PSSMs from databases like PROSITE and BLOCKS. Additionally, the TOPCONS database was used to predict membrane topology for the HPs [36].

### 2.5. Prediction of the Protein Structures and Quality Assessment

Secondary structure prediction was performed using the PSI‐BLAST‐based SOPMA method [23], which utilizes default parameters to analyze the primary sequence. PSIPRED, an artificial neural network‐based algorithm, was also employed to predict the protein sequence′s secondary structures (including beta sheets, alpha helices, and coils) [24]. Results from SOPMA were used to validate findings from the PSIPRED server. Moreover, the three‐dimensional structure of the protein was predicted using the AlphaFold3 server [34], which applies advanced deep learning to accurately model atomic coordinates based on sequence alignments and structural templates. The structure was visualized with the UCSF ChimeraX tool [37]. To evaluate the reliability of the predicted 3D protein structure, we employed SAVES Version 6.0, a meta server that integrates six different programs for thorough validation. This tool assesses stereochemical consistency by analyzing residue geometry and overall structural geometry. Quality was further evaluated using PROCHECK [28], Verify3D [27], QMEAN [22], and ERRAT [26] programs available on the ExPASy server of SWISS‐MODEL Workspace.

### 2.6. Analysis of Virulence Factor

Three bioinformatics tools, VICMpred [30], VirulentPred 2.0 [31], and DeepVF [32], were employed to evaluate the virulence potential of HPs. VICMpred uses SVMs to classify proteins into four functional groups, including virulence factors, by analyzing amino acid and dipeptide composition patterns. VirulentPred 2.0, also based on SVM, specifically distinguishes virulent from nonvirulent proteins by assessing various sequence‐derived features that correlate with pathogenicity. DeepVF takes a more advanced approach by integrating deep learning with traditional feature extraction, combining multiple neural network layers to capture complex, nonlinear patterns from raw protein sequences.

### 2.7. Protein–Protein Interaction (PPI)

The STRING database was employed to analyze PPIs by integrating known and predicted associations, encompassing both physical bindings and functional relationships [33]. It compiles data from multiple sources, including genomic context methods such as gene neighborhood, gene fusion, and gene co‐occurrence, which indicate evolutionary and functional links between proteins. Additionally, it uses conserved coexpression patterns derived from transcriptomic studies, suggesting functional association when genes are coexpressed across different conditions or species.

### 2.8. Prediction of Active and Ligand‐Binding Sites

Accurate identification of ligand‐binding or active sites is crucial for characterizing therapeutic target proteins [44]. To locate these sites, we employed PrankWeb, which implements the P2Rank algorithm [29]. P2Rank uses machine learning to detect binding pockets by clustering surface points based on local geometry, hydrophobicity, and evolutionary conservation (derived from MSAs), yielding a probabilistic ligand ability score. Additionally, we used the ProBiS web server to predict potential ligands [38]. The ProBiS algorithm aligns and superposes whole protein surfaces, surface motifs, and binding sites to detect local structural similarity. It supports pairwise alignment of full structures or user‐defined sites and enables rapid database searches for similar pockets. By comparing local surface geometry, ProBiS identifies analogous binding sites across proteins with divergent folds and without prior site annotations. The predicted ligand structures were retrieved from PubChem [39], a comprehensive and publicly accessible chemical database maintained by the NCBI. PubChem allows chemical structure searches based on compound name, molecular formula, SMILES, InChI, and other identifiers and serves as a primary source for experimentally validated chemical information used in docking studies.

### 2.9. Investigating Molecular Docking Studies

Protein–ligand docking was performed using AutoDock v4.5.6 to identify energetically favorable binding poses [40]. The AlphaFold‐predicted structure of HPs and the ligand predicted by ProBiS were prepared in PDBQT format, which encodes atomic coordinates, partial charges, and solvation parameters. A grid box (126 × 126 × 126 Å) was centered on the identified active site of the protein to encompass potential binding modes. Docking simulations employed the Lamarckian genetic algorithm (GA), an adaptive local search method, with 50 independent runs. The lowest binding energy conformation from the generated ensemble was selected for analysis. Protein–ligand interactions, including hydrophobic contacts and hydrogen bonds, were visualized using the PyMOL server [41] and LigPlot v.2.3 [42].

## 3. Results

### 3.1. Physicochemical Properties and Subcellular Localization

The physicochemical properties of the selected HPs were evaluated using the ProtParam tool and are summarized in (Table 2). The protein EUJ18943.1 consists of 136 amino acids, with a molecular weight of 15,423.48 Da, a theoretical pI of 5.35, and a GRAVY value of −0.300. Its instability index (II) was calculated to be 35.16, classifying it as a stable protein. In comparison, EUJ18676.1 comprises 205 amino acids, with a molecular weight of 24,095.61 Da, a theoretical pI of 5.79, and a GRAVY value of −0.717. The II for EUJ18676.1 was predicted to be 39.15, also indicating stability.

**Table 2:** ProtParam estimates the physicochemical properties of the HPs.

**Descriptions**	**EUJ18943.1**	**EUJ18676.1**
No. of amino acids	136	206
Molecular weight (Da)	15,423.48	24,095.61
Theoretical PI	5.35	5.79
No. of positively charged residues	16	31
No. of negatively charged residues	22	34
No. of atoms	2140	3399
Instability index	35.16	39.15
Aliphatic index	73.90	73.88
Grand average of hydropathicity	−0.300	−0.717
Extinction coefficients	6085	27,390

Further analysis revealed that EUJ18943.1 contains 16 positively charged residues (Arg + Lys) and 22 negatively charged residues (Asp + Glu), while EUJ18676.1 has 31 positively charged and 34 negatively charged residues. The molecular formulas for EUJ18943.1 and EUJ18676.1 were determined to be C_682_H_1058_N_182_O_210_S_8_ and C_1093_H_1700_N_278_O_321_S_7_, respectively. Subcellular localization was predicted using CELLO v2.5 and further validated by PSORTb and PSLpred. All three prediction tools consistently indicated that both proteins are localized in the cytoplasm.

### 3.2. Similarity Detection and Phylogenetic Analysis

A BLASTp search against the nr protein database revealed that EUJ18943.1 exhibits high sequence homology (up to 100% identity) with Immunity protein 48 (Imm48) family immunity proteins from various *Listeria* species (Table 3). Similarly, EUJ18676.1 showed high sequence homology (up to 99% identity) with GyrI‐like domain‐containing protein from *Listeria* species (Table 4). Based on the BLASTp analysis, 10 homologous protein sequences, including the query proteins, were selected for further study. MSA was performed using the CLC Sequence Viewer to identify conserved and variable residues among these homologs (Figure 2). The alignment highlighted several conserved regions, suggesting these areas play important roles in maintaining the structural and functional properties of the proteins. Subsequently, a phylogenetic tree was generated to explore the evolutionary relationships between the sequences (Figure 3). The results showed that EUJ18943.1 is closely related to WP_036072360.1 from *L. aquatica*, sharing a common ancestor. Likewise, EUJ18676.1 and WP_036072677.1 seem to have diverged from a mutual ancestor along with other *L. aquatica* proteins. The scale bar on the phylogenetic tree denotes the degree of sequence divergence, with line segments and numerical values (0.300 for EUJ18943.1 and 0.180 for EUJ18676.1) reflecting the magnitude of genetic variation.

**Table 3:** Similar proteins obtained from nonredundant sequences of EUJ18943.1 HP.

**Description**	**Scientific name**	**Total score**	**Query cover**	**E** **-value**	**Per. ident**	**Accession**
Imm48 family immunity protein	*Listeria aquatica* FSL S10‐1188		100%	9.00e − 96	100.00%	EUJ18943.1
Imm48 family immunity protein	*Listeria aquatica*	283	100%	9.00e − 96	100.00%	WP_036072360.1
Imm48 family immunity protein	*Listeria aquatica*	276	97%	1.00e − 92	100.00%	WP_345818876.1
Imm48 family immunity protein	*Listeria liveliness*	274	100%	2.00e − 92	97.06%	WP_115751713.1
Imm48 family immunity protein	*Listeria valentina*	270	100%	8.00e − 91	94.85%	WP_167628434.1
Imm48 family immunity protein	*Listeria floridensis*	244	100%	2.00e − 80	85.29%	WP_036098337.1
Hypothetical protein MFLO_14132	*Listeria floridensis* FSL S10‐1187	241	100%	3.00e − 79	84.56%	EUJ26361.1
Imm48 family immunity protein	*Listeria costaricensis*	168	100%	2.00e − 50	57.35%	WP_099224602.1
MULTISPECIES: Imm48 family immunity protein	*Listeria* spp.	167	96%	3.00e − 50	61.07%	WP_088810222.1
Imm48 family immunity protein	*Listeria ilorinensis*	162	100%	5.00e − 48	55.15%	WP_239257130.1

**Table 4:** Similar proteins obtained from nonredundant sequences of EUJ18676.1 HP.

**Description**	**Scientific name**	**Total score**	**Query cover**	**E** **-value**	**Per. ident**	**Accession**
GyrI‐like domain‐containing protein	*Listeria aquatica*	423	100%	4.00e − 149	100.00%	EUJ18676.1
GyrI‐like domain‐containing protein	*Listeria aquatica*	423	100%	4.00e − 149	100.00%	WP_036072677.1
GyrI‐like domain‐containing protein	*Listeria aquatica*	414	100%	3.00e − 145	97.57%	WP_244964409.1
Hypothetical protein	*Listeria aquatica*	412	100%	7.00e − 145	97.09%	MBC1520700.1
GyrI‐like domain‐containing protein	*Listeria valentina*	373	100%	3.00e − 129	86.89%	WP_167629442.1
GyrI‐like domain‐containing protein	*Listeria kieliensis*	370	100%	3.00e − 128	86.89%	WP_115752234.1
GyrI‐like domain‐containing protein	*Listeria floridensis*	308	100%	7.00e − 104	69.90%	WP_036098170.1
GyrI‐like domain‐containing protein	*Listeria* sp. ILCC797	295	99%	2.00e − 98	69.46%	WP_088840433.1
GyrI‐like domain‐containing protein	*Listeria goaensis*	295	99%	2.00e − 98	69.46%	WP_088825250.1
MULTISPECIES: GyrI‐like domain‐containing protein	Unclassified *Listeria*	294	99%	4.00e − 98	68.97%	WP_088808777.1

Figure 2Multiple sequence alignment of the hypothetical proteins (a) EUJ18943.1 and (b) EUJ18676.1. The figure was generated using CLC Sequence Viewer Version 8.

**Figure:**
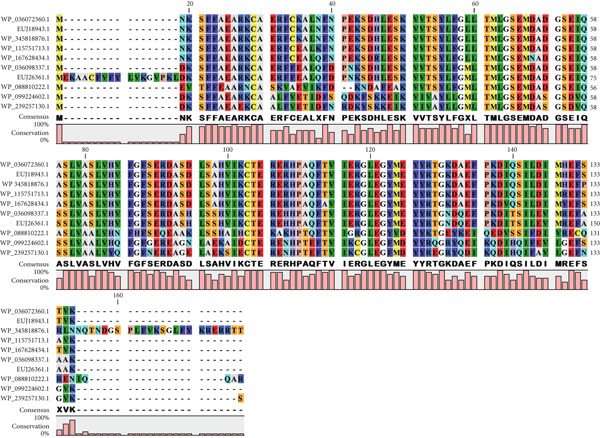
(a)

**Figure:**
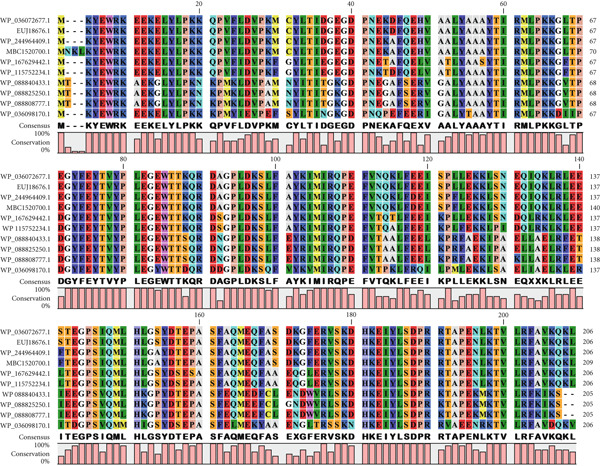
(b)

Figure 3The phylogenetic tree with accurate distance from target proteins (a) EUJ18943.1 and (b) EUJ18676.1. The tree was generated using CLC Sequence Viewer Version 8.

**Figure:**
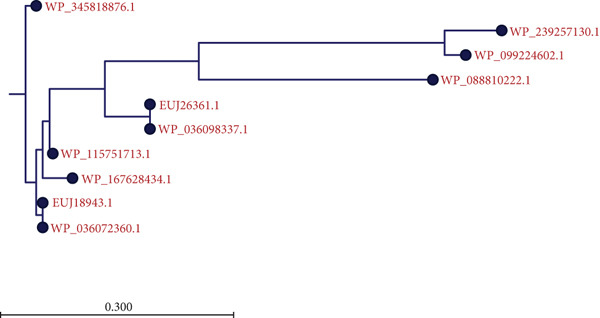
(a)

**Figure:**
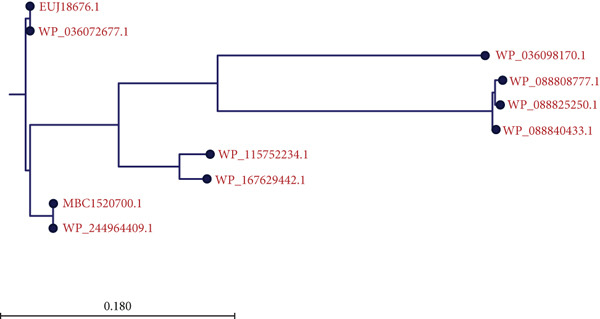
(b)

### 3.3. Functional Annotation of Hypothetical Proteins

The functional annotation and conserved domain analysis of the HPs EUJ18943.1 and EUJ18676.1 were performed using multiple bioinformatics tools, including CDD Search, Pfam, InterProScan, HMMER, and the MOTIF search tool. For EUJ18943.1, the NCBI‐CD‐Search predicted the presence of an Imm48 domain, characterized by an all‐helical fold and a conserved HRG motif. This protein is typically associated with heterogeneous polyimmunity loci involved in polymorphic toxin systems. The predicted domain spans residues 5–128 with a highly significant e‐value of 3.99e − 37. This prediction was corroborated by InterProScan and Pfam, both identifying the same domain boundaries and e‐value. Additionally, HMMER analysis detected the Imm48 protein domain spanning residues 5–130, with an e‐value of 2.1e − 35. The MOTIF server further supported these findings with a matching domain prediction and an independent e‐value of 2.2e − 35. For EUJ18676.1, the NCBI‐CD‐Search identified a cyclopropanoid cyclopropyl hydrolase (CCH) domain, which belongs to the GyrI‐like superfamily. These domains are known to catalyze the hydrolysis of DNA‐alkylating agents such as yatakemycin (YTM) and CC‐1065, thereby conferring cellular protection. The domain was mapped from residues 1–205 with an e‐value of 1.24e − 96. InterProScan and Pfam consistently predicted the presence of the GyrI‐like domain from residues 5–129 with an e‐value of 5.3e − 32. HMMER further confirmed the GyrI‐like small molecule‐binding domain spanning residues 17–203 with an e‐value of 2.7e − 10. Similarly, the MOTIF server identified the same domain with an e‐value of 2.7e − 10.

### 3.4. Prediction of Protein Structures and Quality Assessment

The secondary structure of the target HPs EUJ18943.1 and EUJ18676.1 was predicted using SOPMA and PSIPRED tools. For EUJ18943.1, SOPMA estimated 94 residues (69.12%) as alpha‐helices, 39 residues (28.68%) as random coils, and three residues (2.21%) as extended strands. PSIPRED analysis corroborated these results, predicting 105 residues (77.2%) as alpha‐helices and 31 residues (22.8%) as random coils (Figure 4). In contrast, for EUJ18676.1, SOPMA predicted 69 residues (33.5%) as alpha‐helices, 88 residues (42.7%) as random coils, and 49 residues (23.8%) as extended strands. PSIPRED analysis similarly indicated a predominance of alpha‐helices (293 residues, 81.7%) and 66 residues (18.3%) as random coils (Figure 5).

Figure 4Predicted secondary structure of HP EUJ18943.1. (a) The SOPMA server shows the helix, sheet, and coil structure. (b) The PSIPRED server predicts the secondary structure of the HP. The ash color depicts coil structures, and the pink represents helix structure.

**Figure:**
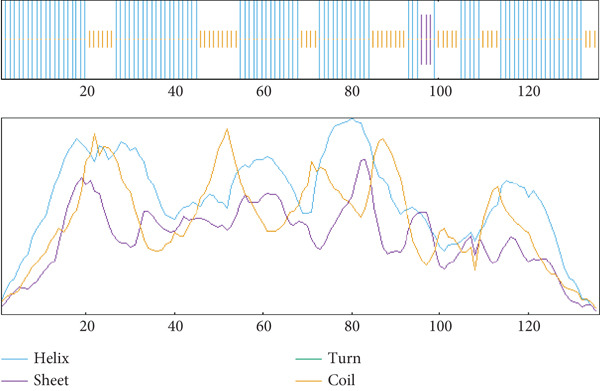
(a)

**Figure:**
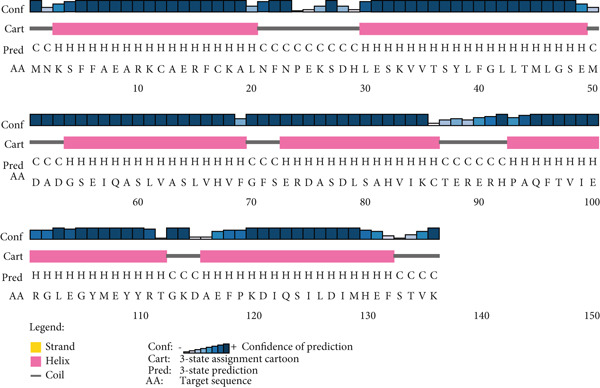
(b)

Figure 5Predicted secondary structure of HP EUJ18676.1. (a) The SOPMA server shows the helix, sheet, and coil structure. (b) The PSIPRED server predicts the secondary structure of the HP. The ash color depicts coil structures, and the pink represents helix structure.

**Figure:**
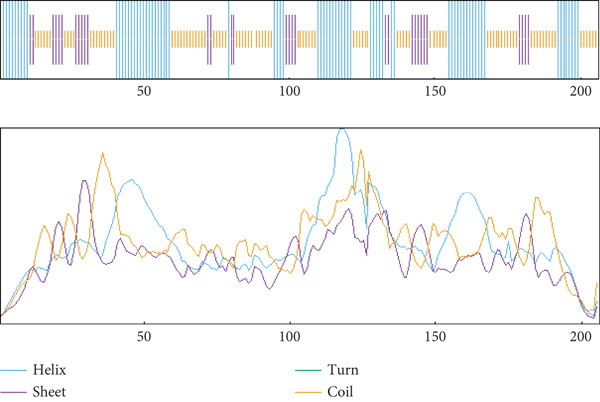
(a)

**Figure:**
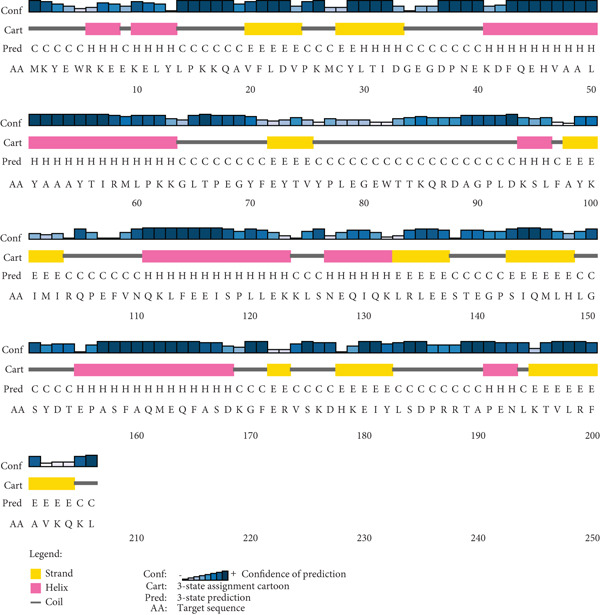
(b)

3D structural models of both proteins were generated using the AlphaFold3 server (Figure 6). The predicted model for EUJ18943.1 exhibited a predicted template modeling (pTM) score of 0.87, indicating a high level of confidence in the global fold of the monomeric structure. Although slightly within the “gray zone” (0.6–0.8), scores above 0.5 generally imply structural similarity to experimentally determined conformations. For EUJ18676.1, the predicted pTM score was 0.94, reflecting even higher confidence in the predicted structure.

Figure 6Predicted 3D structure of the hypothetical protein using AlphaFold Server. (a) Structure belong to EUJ18943.1 protein. (b) Structure belong to EUJ18676.1. The structures were visualized using UCSF ChimeraX tool.

**Figure:**
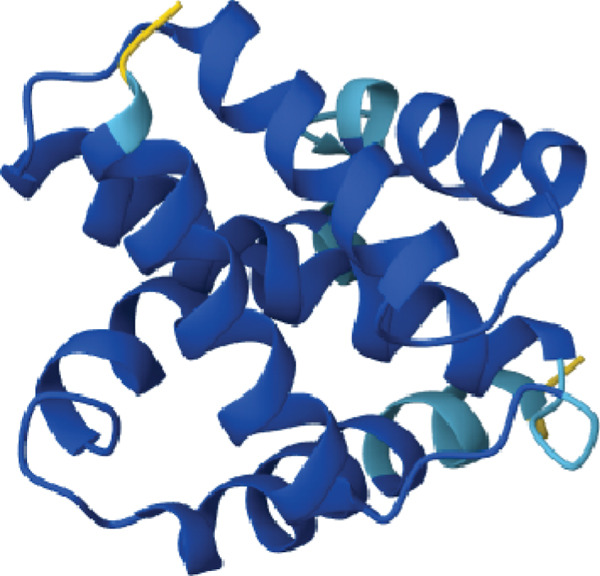
(a)

**Figure:**
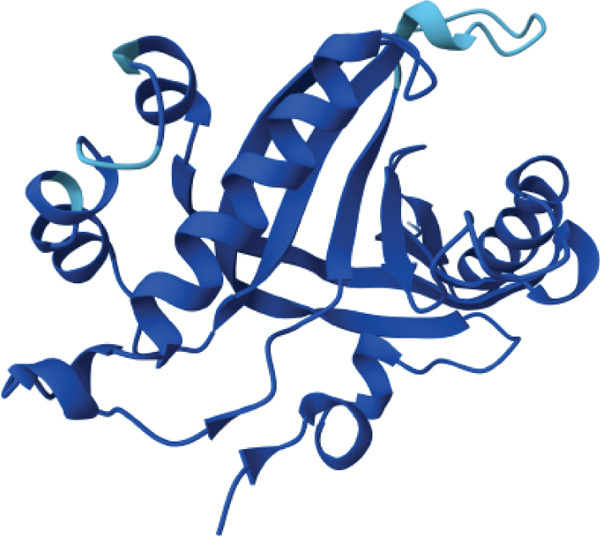
(b)

The structural integrity and quality of the predicted models were evaluated using the SAVES v6.0 meta server, which integrates multiple validation tools including PROCHECK, ERRAT, Verify3D, and QMEAN. For EUJ18943.1, PROCHECK analysis showed 95.2% of residues within the most favored regions and 4.8% in additionally allowed regions of the Ramachandran plot (Table 5 and Figure 7a), indicating excellent stereochemical quality. ERRAT yielded a quality factor of 94.56, surpassing the 90% benchmark typically associated with high‐resolution crystal structures. Verify3D assessment confirmed that 83.09% of residues had acceptable 3D–1D profile scores (≥ 0.2). For EUJ18676.1, PROCHECK revealed 97.2% of residues in the most favored regions and 2.8% in allowed regions, again reflecting excellent geometry (Figure 7b). ERRAT provided a quality factor of 91.41, and Verify3D indicated that 99.51% of residues achieved acceptable profile scores, further supporting the model′s reliability. QMEANDisCo global scores were 0.64 ± 0.07 and 0.72 ± 0.06 for EUJ18943.1 and EUJ18676.1, respectively (Figure 7c,d). These scores suggest moderate confidence in the predicted models and are consistent with monomeric structural features as predicted.

**Table 5:** Ramachandran plot statistics of the target proteins.

**Statistics**	**EUJ18943.1**	**EUJ18676.1**
Residues in most favored regions [A, B, L]	118 (95.2%)	176 (97.2%)
Residues in additional allowed regions [a, b, l, p]	6 (4.8%)	5 (2.8%)
Residues in generously allowed regions [~a, ~b, ~l, ~p]	0 (0.0%)	0 (0.0%)
Residues in disallowed regions	0 (0.0%)	0 (0.0%)
Number of nonglycine and nonproline residues	124 (100.0%)	181 (100.0%)
Number of end‐residues (excl. Gly and Pro)	2	2
Number of glycine residues (shown as triangles)	7	9
Number of proline residues	3	14
Total number of residues	136	206

Figure 7Evaluation of model quality. (a, b) Structural validation of the predicted models for proteins EUJ18943.1 and EUJ18676.1, respectively, using Ramachandran plots generated by the PROCHECK server. Most residues are located within the most favored and additionally allowed regions, indicating good stereochemical quality. (c) QMEAN *Z*‐score analysis of EUJ18943.1 yielded a score of 0.64 ± 0.07, suggesting an acceptable model quality within the range of experimentally determined protein structures. (d) The QMEAN *Z*‐score for EUJ18676.1 was 0.72 ± 0.06, further supporting the reliability of the predicted model.

**Figure:**
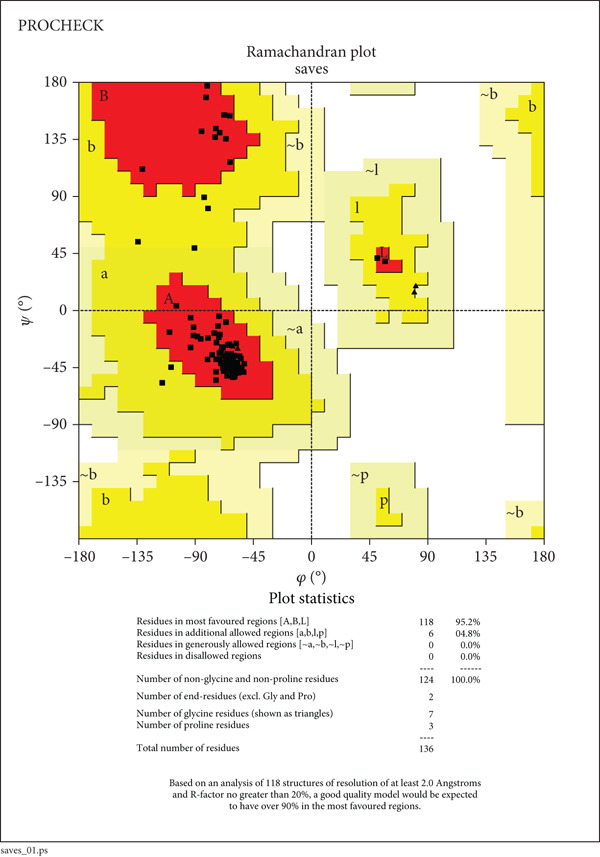
(a)

**Figure:**
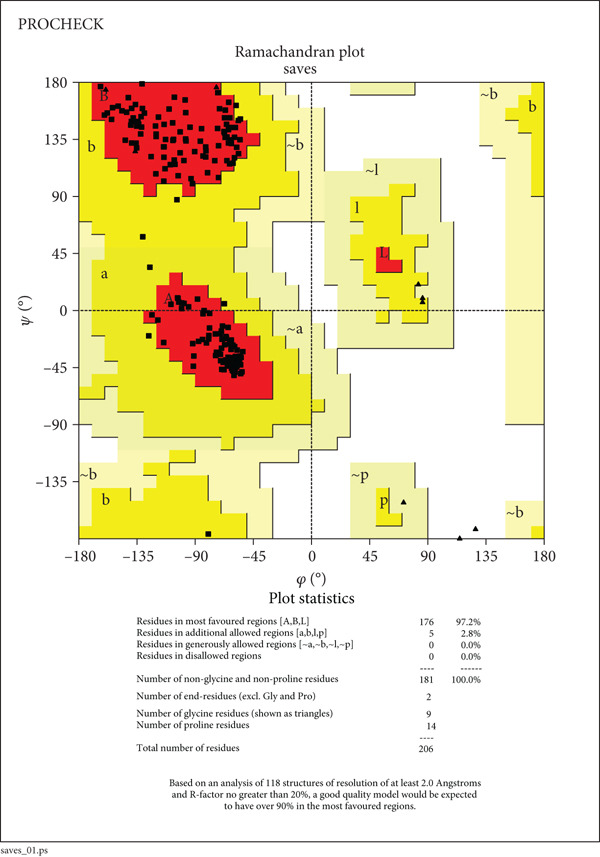
(b)

**Figure:**
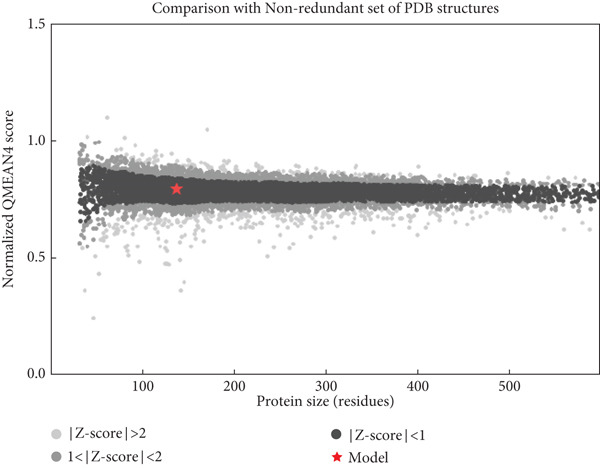
(c)

**Figure:**
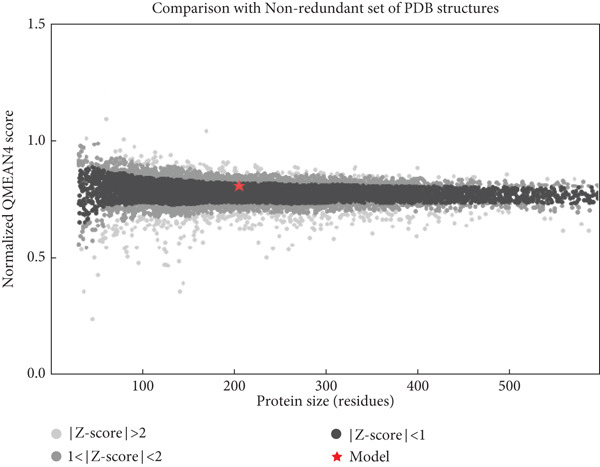
(d)

### 3.5. Prediction of Virulence Factor

The virulence potential of the proteins EUJ18943.1 and EUJ18676.1 from *L. aquatica* FSL S10‐1188 was evaluated using three bioinformatics tools: VICMpred, VirulentPred, and DeepVF. VICMpred, which applies an SVM algorithm based on amino acid patterns and dipeptide composition, classified EUJ18943.1 as a metabolism‐related protein with a score of 1.411. EUJ18676.1, in contrast, was categorized under the cellular process class with a score of 0.8848. VirulentPred uses a bilayer cascade SVM model coupled with fivefold cross‐validation, achieving a reported prediction accuracy of 81.8%. According to VirulentPred, both proteins were identified as potential virulence factors. Similarly, DeepVF analysis yielded virulent scores of 0.871 for EUJ18943.1 and 0.882 for EUJ18676.1, further supporting the potential virulence of these proteins.

### 3.6. Active Site Prediction of Hypothetical Proteins

The active site prediction for the HPs EUJ18943.1 and EUJ18676.1 was carried out using the PrankWeb server. The preprocessed PDB structures, generated by the AlphaFold3 server, were submitted to PrankWeb for analysis. This tool identifies and ranks potential ligand‐binding pockets based on pocket scores, which indicate the statistical likelihood of functional relevance, and higher scores correspond to greater confidence. For EUJ18943.1, PrankWeb predicted a total of four putative binding pockets: Pocket 1 exhibited the highest confidence, with a pocket score of 4.98, a probability score of 0.236, and consisted of 12 amino acid residues; Pocket 2 had a pocket score of 2.96, a probability score of 0.097, and included 10 residues; Pocket 3 presented a pocket score of 2.81, a probability score of 0.088, and comprised nine residues; and Pocket 4, with the lowest confidence, had a pocket score of 0.97, a probability score of 0.006, and consisted of eight residues (Figure 8a). In contrast, EUJ18676.1 exhibited only two predicted binding pockets: Pocket 1 showed the highest confidence, with a pocket score of 31.82, a probability score of 0.926, and contained 33 amino acid residues; and Pocket 2 had a score of 0.92, a probability score of 0.005, and included only three residues (Figure 8b). These predictions suggest that both proteins possess well‐defined ligand‐binding regions, with EUJ18676.1 exhibiting a particularly high‐confidence primary pocket, potentially indicative of a biologically significant active site.

Figure 8Ligand‐binding sites of the HPs. (a) The structure of EUJ18943.1 revealed four distinct ligand‐binding pockets. (b) The predicted structure of EUJ18676.1 showed two binding pockets. Different colors represent different pockets: red (Pocket 1), yellow (Pocket 2), orange (Pocket 3), and blue (Pocket 4). The structures and binding pockets were predicted using the PrankWeb server.

**Figure:**
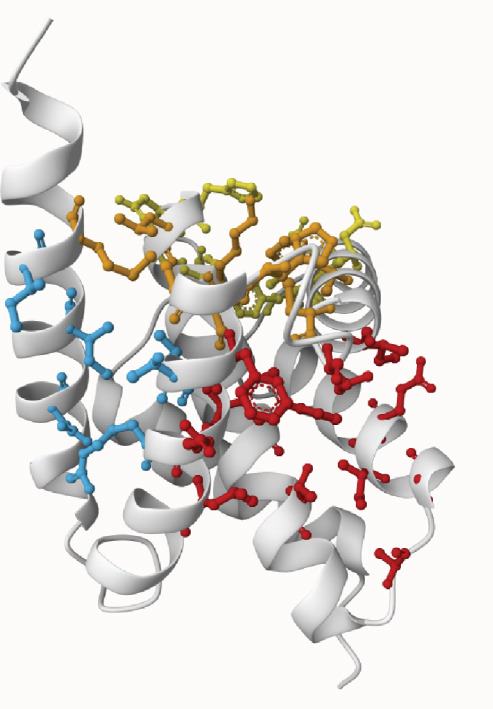
(a)

**Figure:**
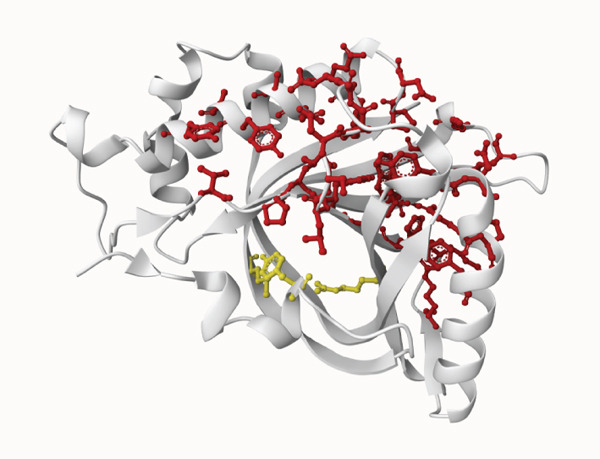
(b)

### 3.7. PPI Network

STRING is dedicated to offering dependable protein interaction predictions with quantified confidence levels to aid interpretation. Its predictive power lies in its ability to integrate various types of evidence and benchmark them against a standard reference set, usually KEGG pathways. Subsequently, the scores from different evidence channels are consolidated to produce a final confidence score for each predicted association [45, 46]. In this analysis, STRING Version 12.0 was employed to investigate the potential functional interaction networks of the hypothetical proteins EUJ18943.1 and EUJ18676.1. For EUJ18943.1, the resulting network comprised five nodes and eight edges, with an average node degree of 3.2 and a PPI enrichment *p* value of 0.049 (Figure 9a). In contrast, the interaction network for EUJ18676.1 revealed nine nodes and 22 edges, with a higher average node degree of 4.89 and a highly significant PPI enrichment *p*‐value of 3.86e − 05 (Figure 9b), indicating strong functional connectivity. The predicted functional partners for both proteins are detailed in (Table 6).

Figure 9STRING 12.0 analysis of protein–protein interaction (PPI) networks for the hypothetical proteins. (a) The interaction network of EUJ18943.1 consists of five nodes and eight edges. (b) The interaction network of EUJ18676.1 comprises nine nodes and 22 edges.

**Figure:**
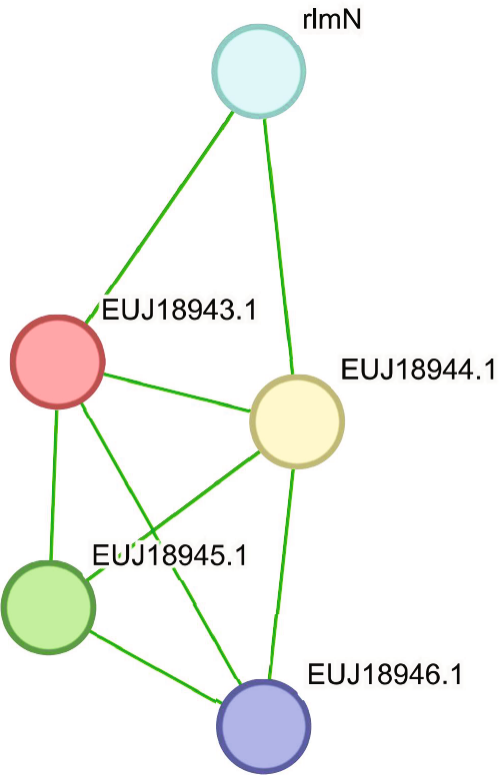
(a)

**Figure:**
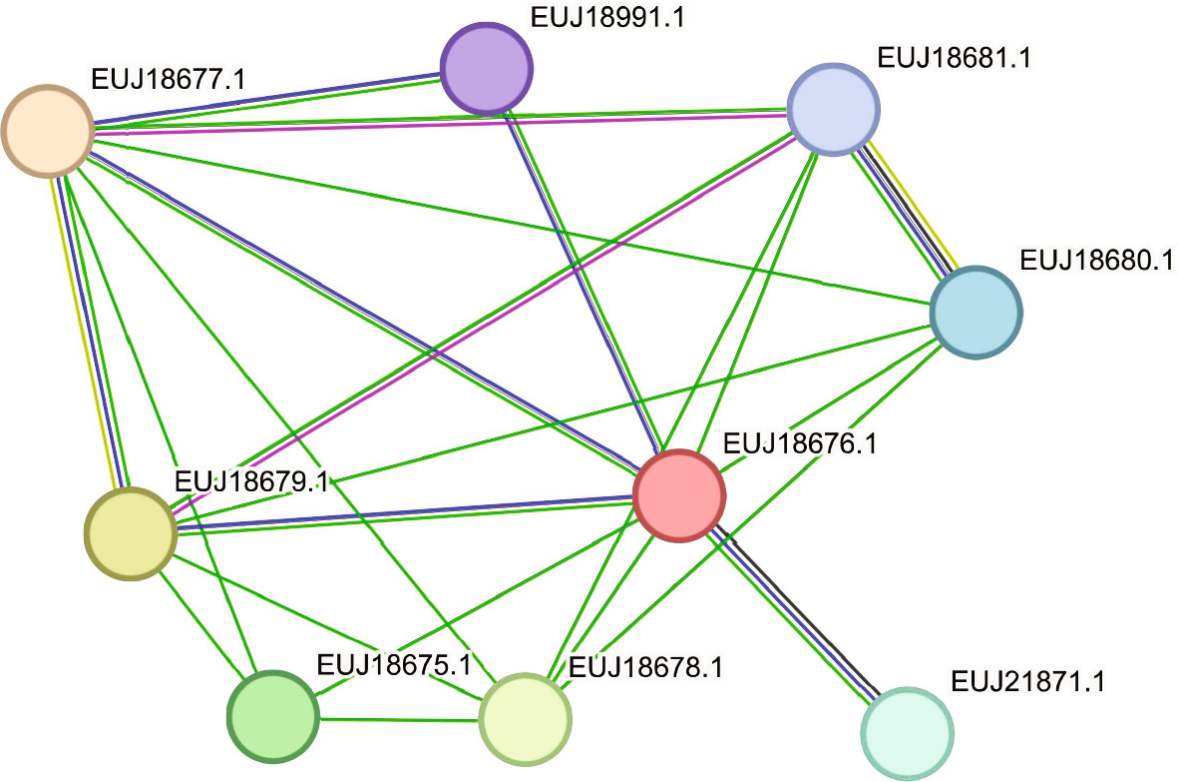
(b)

**Table 6:** The predicted functional partners of the HPs using the STRING database.

**Protein**	**Description**	**Score**
EUJ18943.1		
EUJ18944.1	Hypothetical protein	0.773
EUJ18945.1	Hypothetical protein; COG2060 K + ‐transporting ATPase, A chain	0.598
rlmN	Ribosomal RNA large subunit methyltransferase N; RlmN family	0.415
EUJ18946.1	COG0656 aldo/keto reductases, related to diketogulonate reductase	0.41
EUJ18676.1		
EUJ18677.1	COG4978 transcriptional regulator, effector‐binding domain/component	0.866
EUJ18679.1	COG0789 predicted transcriptional regulators	0.832
EUJ18678.1	Hypothetical protein	0.773
EUJ18675.1	Heat shock protein, Hsp20 family; COG0071 molecular chaperone (small heat shock protein)	0.574
EUJ21871.1	Hypothetical protein	0.502
EUJ18680.1	Hypothetical protein	0.445
EUJ18681.1	Hypothetical protein	0.425
EUJ18991.1	TfoX domain‐containing protein; COG3070 regulator of competence‐specific genes	0.416

### 3.8. Ligand‐Binding Interactions and Molecular Docking

The ProBiS web server was utilized to predict potential ligand‐binding sites for the HPs. For EUJ18943.1, kanamycin A emerged as the top‐ranked ligand with a confidence score of 2.32, while streptomycin was identified for EUJ18676.1 with a confidence score of 2.10. The 3D structures of these ligands were retrieved from the PubChem database. To evaluate the binding orientation and stability within the predicted active sites, molecular docking simulations were conducted using AutoDock v4.5.6. A total of 50 independent docking runs were performed, and the conformations with the lowest binding free energy were selected as the most favorable. The best docked poses exhibited binding free energies (*Δ* *G*) of −7.17 kcal/mol for EUJ18943.1 and −4.30 kcal/mol for EUJ18676.1. Postdocking analysis using PyMOL and LigPlot v2.3 revealed key interacting residues within the binding pockets. For EUJ18943.1, the residues Asn2, Lys3, Met50, Asp51, and Glu56 were involved in ligand interactions (Figure 10) For EUJ18676.1, the binding interface included Ser126, Gln129, Lys132, Asp42, and Glu45 (Figure 11). These residues likely contribute to ligand‐binding through hydrogen bonding and hydrophobic interactions, supporting the predicted binding modes and highlighting the potential functional relevance of these hypothetical proteins.

Figure 10Molecular docking simulations of EUJ18943.1 with kanamycin A using AutoDock v4.5.6. (a) Cartoon representation of the binding site for EUJ18943.1 and kanamycin A. (b) LigPlot v2.3 diagram illustrating the hydrophobic interactions between the protein and ligand complex. (c) Surface representation of the docking binding pocket for the protein–ligand complex. (d) Detailed view of the ligand‐binding site and the interacting residues. (a, c, d) were generated using PyMOL software, while (b) was created with LigPlot v2.3.

**Figure:**
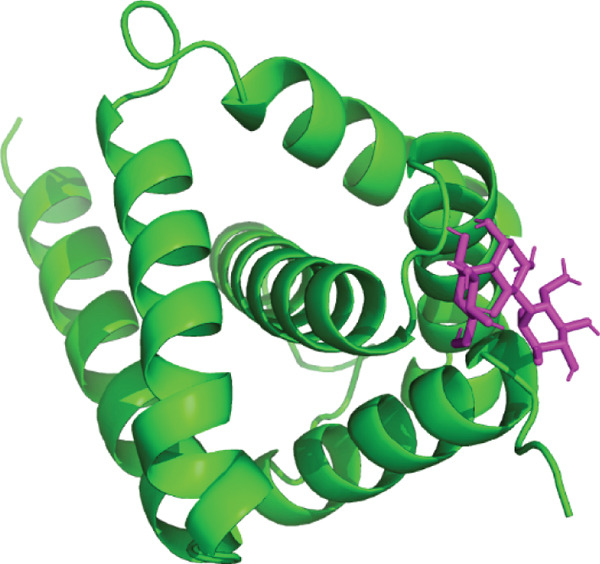
(a)

**Figure:**
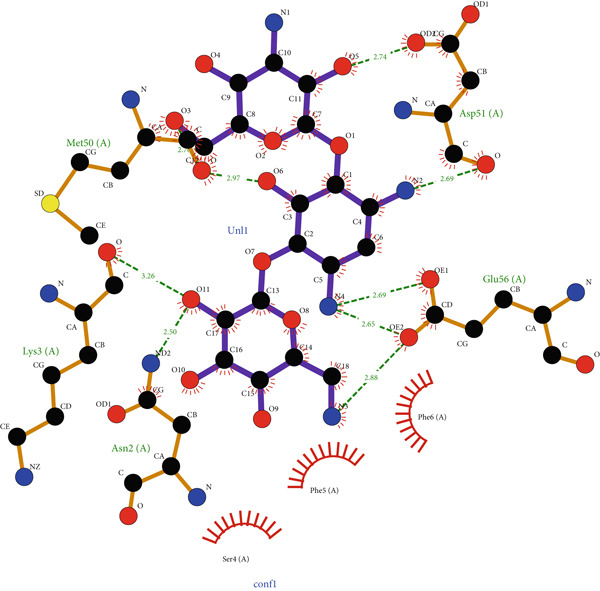
(b)

**Figure:**
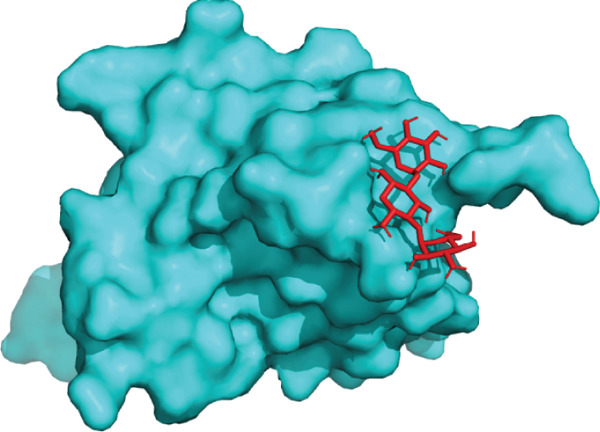
(c)

**Figure:**
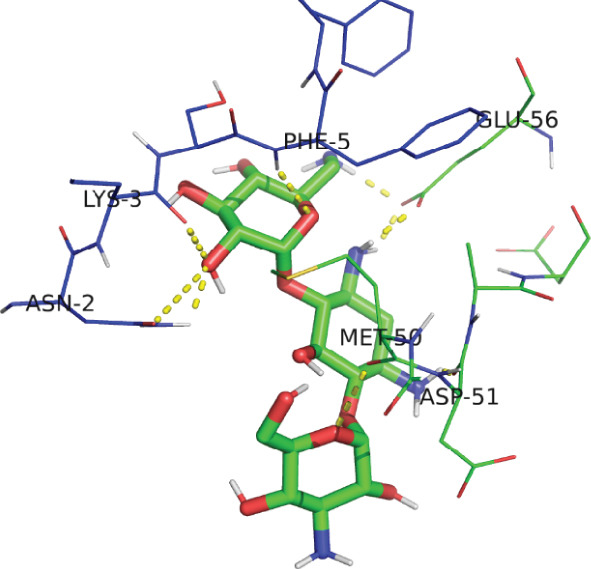
(d)

Figure 11Molecular docking simulations of EUJ18676.1 with streptomycin using AutoDock v4.5.6. (a) Cartoon representation of the binding site for EUJ18676.1 and streptomycin. (b) LigPlot v2.3 diagram illustrating the hydrophobic interactions between the protein and ligand complex. (c) Surface representation of the docking binding pocket for the protein–ligand complex. (d) Detailed view of the ligand‐binding site and the interacting residues. (a, c, d) were generated using PyMOL software, while (b) was created with LigPlot v2.3.

**Figure:**
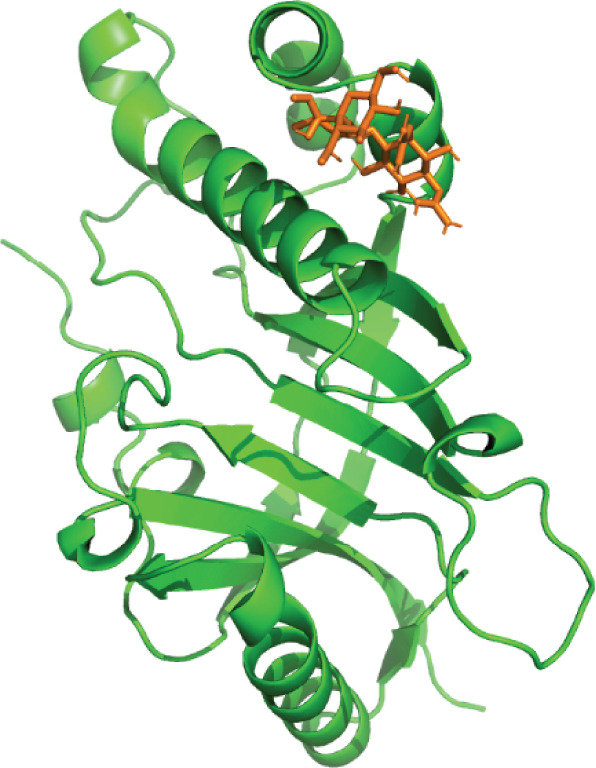
(a)

**Figure:**
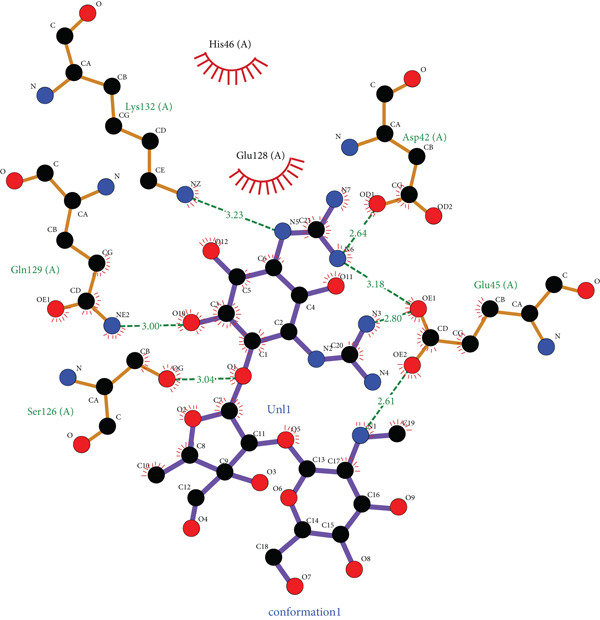
(b)

**Figure:**
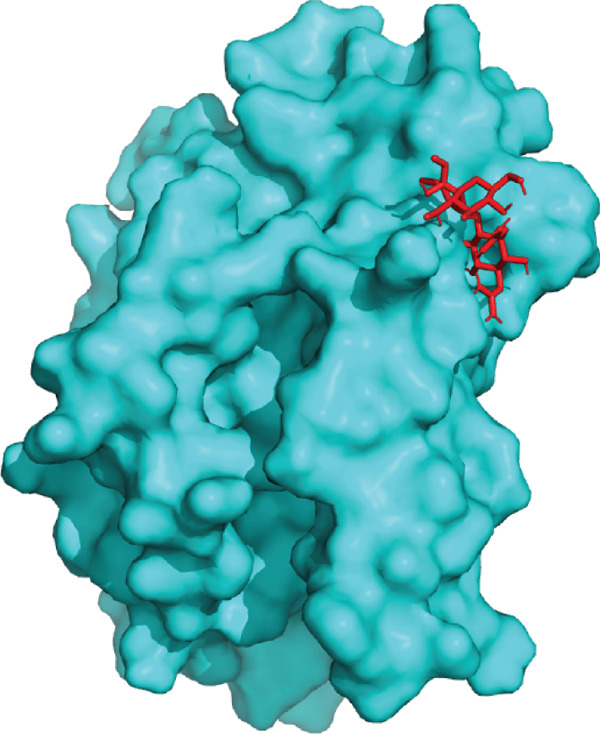
(c)

**Figure:**
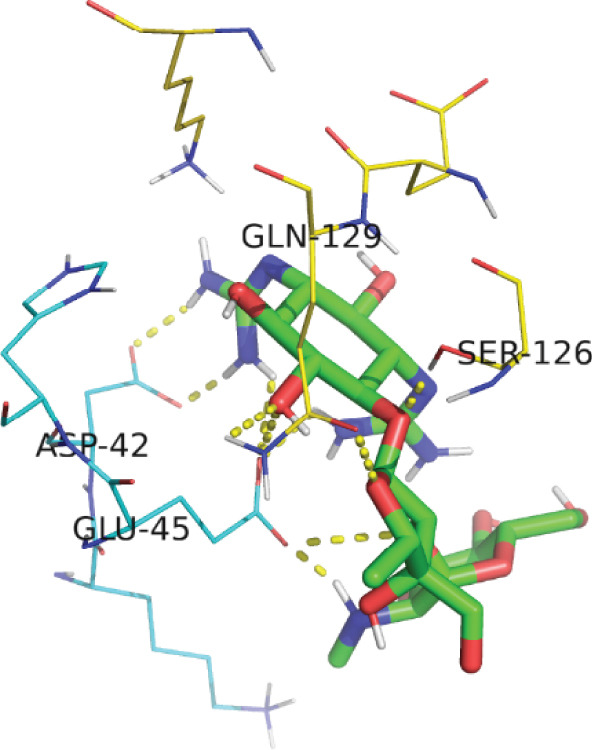
(d)

## 4. Discussion

*L. aquatica* is notable among *Listeria* species for its inability to grow at low temperatures and its distinct ability to ferment maltose and D‐tagatose. This combination of traits sets it apart, as most *Listeria* species adapt to soil or food‐associated environments, whereas *L. aquatica*′s features are thought to reflect evolutionary adaptation to freshwater habitats, typically fresh running water rather than terrestrial sources [47, 48]. Genomic analyses show that *L. aquatica* possesses a genome size ranging from about 2.6 to 2.7 Mbp, with a GC content around 39.6%, which is similar to other members of the genus [48, 49]. This genome encodes roughly 2855–3851 protein‐coding genes, including an abundance of genes associated with stress response and nutrient uptake traits that help the organism survive under fluctuating aquatic conditions, such as nutrient limitation or variable osmotic stresses [49].

Out of 2789 predicted genes, 919 were initially identified as hypothetical proteins. After filtering for sequence length (> 50 residues) to exclude unstable or nonglobular sequences, 883 were retained. Additional assessment using ProtParam for protein stability and consensus subcellular localization tools (CELLO, PSORTb, and PSLpred) further narrowed the pool to 297 HPs (611 stable proteins minus 314 with inconclusive localization). This rigorous sequential filtering ensures that downstream analyses focus on the most promising, well‐characterized HPs, providing higher confidence in subsequent functional predictions. Such silico screening is a widely accepted approach to prioritize targets for experimental follow‐up [10, 50, 51].

The HPs EUJ18943.1 and EUJ18676.1 comprise 136 and 206 amino acid residues, respectively, and possess acidic theoretical pIs. Both proteins are predicted to be relatively stable, with IIs below 40, suggesting a good likelihood of structural integrity under physiological conditions. Their negative GRAVY scores indicate a hydrophilic nature, consistent with possible roles in aqueous or extracellular environments [16]. Additionally, their extinction coefficients are within a measurable range, supporting their suitability for downstream biochemical and spectrophotometric characterization. The extinction coefficients at 280 nm, calculated using the ProtParam tool, were estimated to be 6085–5960 M^−1^ cm^−1^ for EUJ18943.1 and 27390 M^−1^ cm^−1^ for EUJ18676.1, with the latter showing a consistent value across both calculations. These coefficients reflect the presence of aromatic amino acids tryptophan, tyrosine, and cysteine which are primarily responsible for ultraviolet absorbance at this wavelength [15].

Consistent subcellular localization predictions from CELLO, PSORTb, and PSLpred support the cytoplasmic localization of both proteins, enhancing the validity of the finding. CELLO employs a two‐level SVM based on amino acid composition and physicochemical features to predict localization with high accuracy [16]. PSORTb integrates homology‐based inference, motif detection, and machine learning to classify proteins into refined localization categories, specifically optimized for prokaryotic proteins [17]. PSLpred utilizes a neural network trained on curated experimental data to predict bacterial protein localization [18]. The predicted cytoplasmic localization aligns well with the physicochemical characteristics typically associated with cytoplasmic proteins. These proteins generally exhibit hydrophilic surfaces, acidic to neutral pIs, and moderate molecular stability, which are suitable for functioning within the highly aqueous and enzymatically active cytoplasmic environment. Cytoplasmic proteins usually lack signal peptides and transmembrane domains, further supporting their intracellular localization [34].

BLASTp analysis revealed that the protein sequence EUJ18943.1 shares up to 100% sequence identity with members of the Imm48 protein family across multiple *Listeria* species. This high level of conservation suggests that EUJ18943.1 performs similar structural and functional roles as other Imm48 proteins. Imm48 is a small, highly conserved immunity protein widely distributed among *Listeria* spp., known to confer protection against bacteriocin toxins produced by the same strain or closely related bacteria [52]. Similarly, EUJ18676.1 displays up to 99% sequence identity with GyrI‐like domain‐containing proteins from *Listeria* spp., indicating a likely functional similarity. GyrI‐like proteins constitute a family of prokaryotic proteins with established roles in detoxification and resistance mechanisms. Notably, this family includes Lin2189 from *Listeria innocua* (UniProt ID: Q929T5), which functions as a CCH. These enzymes catalyze the hydrolysis of highly toxic DNA‐alkylating agents, such as YTM and CC‐1065, thereby protecting bacterial cells from genotoxic stress [53].

The structural characterization of HP EUJ18943.1 and EUJ18676.1 provides valuable insight into their potential biological functions. Secondary structure predictions using SOPMA and PSIPRED revealed a predominantly alpha‐helical structure for both proteins, albeit to differing extents. EUJ18943.1 exhibited a higher alpha‐helical content (69.12% by SOPMA; 77.2% by PSIPRED) (Figure 4), suggesting compact and potentially globular architecture. In contrast, EUJ18676.1 displayed a more diverse secondary structure profile, with SOPMA indicating a balance among alpha‐helices (33.5%), random coils (42.7%), and extended strands (23.8%), whereas PSIPRED predicted a strong alpha‐helical dominance (81.7%) (Figure 5). Secondary structure prediction offers insights into protein folding, guiding functional inference, structural modeling, and identification of interaction sites. It also aids in comparing conserved regions across protein families, supporting evolutionary and functional analyses [54, 55].

Tertiary structures generated by AlphaFold3 showed high pTM scores of 0.87 for EUJ18943.1 and 0.94 for EUJ18676.1 (Figure 6), indicating substantial confidence in the global fold accuracy, particularly for EUJ18676.1. AlphaFold3 represents a major advancement in computational structural biology, offering highly accurate and reliable protein structure predictions that closely align with experimentally determined models [56]. This enables robust functional annotation and structural insight, particularly for HPs with unknown homologs or experimentally resolved structures, thereby accelerating discoveries in protein function, interaction, and drug targeting through purely in silico approaches. The structural models were further validated through the SAVES v6.0 meta‐server, incorporating key quality assessment tools. For EUJ18943.1, the Ramachandran plot analysis by PROCHECK demonstrated that 95.2% of residues were in the most favored regions, while ERRAT and Verify3D scores exceeded quality thresholds, supporting the reliability of the model. Similarly, EUJ18676.1 showed exceptional stereochemical quality, with 97.2% of residues in the most favored regions and a higher Verify3D pass rate of 99.51%, indicative of excellent 3D–1D compatibility.

The QMEANDisCo global scores (0.64 ± 0.07 for EUJ18943.1 and 0.72 ± 0.06 for EUJ18676.1) (Figure 7c,d), respectively, suggest moderate to high model reliability, consistent with the high‐resolution quality indicated by ERRAT and the favorable geometry in the Ramachandran plots. Collectively, these validation methods confirm that the predicted structures of EUJ18943.1 and EUJ18676.1 are structurally reliable, realistic, and biologically relevant. Establishing the accuracy of structural models is a critical prerequisite for confident functional annotation, experimental design, and downstream applications such as drug targeting or protein engineering [57, 58].

VICMpred classified EUJ18943.1 as metabolism‐related and EUJ18676.1 as involved in cellular processes, implying they contribute to fundamental bacterial functions that could support pathogenicity. VirulentPred′s identification of both as virulence factors with 81.8% accuracy, alongside DeepVF’s high virulent scores (around 0.87–0.88), further strengthens the evidence that these proteins might contribute directly to bacterial virulence. Machine learning and deep learning–based bioinformatics tools improve virulence factor prediction by modeling complex biological patterns and learning from high‐quality datasets, outperforming traditional similarity‐ or motif‐based methods in accuracy and generalization [59, 60].

The active site prediction using PrankWeb revealed multiple ligand‐binding pockets for both hypothetical proteins. EUJ18943.1 has four predicted pockets with moderate confidence scores, the top pocket showing a pocket score of 4.98 and involving 12 residues suggesting potential functional sites. In contrast, EUJ18676.1 presented two pockets, with the primary pocket displaying a very high confidence (pocket score 31.82, probability 0.926) and encompassing 33 residues, indicating a strong likelihood of a biologically relevant active site. Predicting the active site of a protein is important to identify the specific region where the protein interacts with ligands, substrates, or other molecules essential to its biological function [61].

The ligand‐binding interaction and molecular docking results using ProBiS and AutoDock provide important functional insights into the HPs EUJ18943.1 and EUJ18676.1. ProBiS identifies potential ligands by comparing 3D surface amino acid patterns with known proteins in the PDB, allowing prediction of biologically relevant small molecules that may bind the target proteins [62]. For EUJ18943.1, kanamycin A was the top ligand predicted, and for EUJ18676.1, streptomycin ranked highest, both supported by confidence scores around 2.1–2.3, indicating credible predictions based on structural similarity to known binding sites. This is the first report linking these HPs from *L. aquatica* with potential antibiotic interactions, which could imply roles in drug recognition or resistance mechanisms.

Molecular docking with AutoDock refines these predictions by simulating ligand‐binding orientations and energetics inside the predicted pockets. The observed binding free energies (*Δ* *G* of −7.17 and −4.30 kcal/mol) suggest favorable and stable ligand–protein interactions. The identified key residues within the binding sites such as Asn2, Lys3, Met50, Asp51, and Glu56 for EUJ18943.1 and Ser126, Gln129, Lys132, Asp42, and Glu45 for EUJ18676.1 likely contribute through hydrogen bonds and hydrophobic contacts, reinforcing the functional relevance of these sites in ligand recognition. Molecular docking translates structural predictions into plausible biochemical functions by linking the protein′s 3D conformation to its potential interaction with biologically relevant molecules, strongly supporting the HP′s functional annotation [63].

This study provides novel insights into the molecular and functional attributes of the understudied environmental species *L. aquatica*. By characterizing two HPs with potential roles in stress adaptation and virulence, it enhances our understanding of bacterial survival mechanisms in aquatic ecosystems. Additionally, the study presents a robust computational pipeline for the systematic annotation of uncharacterized proteins, demonstrating its utility for large‐scale genomic analysis. Future research should prioritize experimental validation to confirm the predicted functions and biological relevance of these proteins.

## 5. Conclusion

In this study, we conducted an in‐depth bioinformatics analysis of two hypothetical proteins, EUJ18943.1 and EUJ18676.1, derived from the bacterium *L. aquatica* FSL S10‐1188. Various bioinformatics tools were employed to investigate their physicochemical and functional properties. Sequence similarity searches, coupled with phylogenetic analysis, revealed that EUJ18943.1 is classified within the Imm48 immunity protein domain, while EUJ18676.1 aligns with the GyrI‐like detoxification protein family. Both proteins share significant evolutionary relationships with homologous proteins found in other *Listeria* species, indicating conserved functional roles within the genus.

The identification of HPs as a cytoplasmic protein suggests its potential suitability for inclusion in vaccine design. Moreover, the molecular docking analysis performed herein provides a preliminary foundation for future in silico vaccine development studies. This work may serve as a valuable resource for researchers pursuing similar studies in the field.

Our findings contribute to the understanding of the structural and functional characteristics of proteins with unknown functions, offering insights that can inform future experimental investigations. Validation of the predicted functions and further exploration of the proteins′ potential as therapeutic targets in *L. aquatica* are warranted. Continued experimental research is essential to fully elucidate their biological roles and significance, particularly in the context of the bacterium′s pathogenic potential.

## Conflicts of Interest

The authors declare no conflicts of interest.

## Author Contributions

**Mutaz Mohammed Abdallah:** writing – review and editing, writing – original draft, validation, software, formal analysis, conceptualization. **Ruaa Abdalla Ibrahim Suliman:** writing – review and editing, visualization, formal analysis, conceptualization. **Yousra Tagelsir Ahmed:** writing – original draft, validation, formal analysis. **Mawada Yahia:** conceptualization, supervision.

## Funding

No funding was received for this manuscript.

## Supporting Information

Additional supporting information can be found online in the Supporting Information section.

## Supporting information


**Supporting Information 1** Data S1: The physicochemical properties of these selected HPs were assessed using the ProtParam tool.

**Supporting Information 2** Data S2: Subcellular localization investigation of the HPs with three computational tools: CELLO v2.5, PSORTb v3.0.3, and PSLpred.

**Supporting Information 3** Data S3: Functional domain annotation using the NCBI Conserved Domain Search (CD‐Search), InterProScan5, Pfam 37.1, HMMER, and MOTIF.

## Data Availability

This work did not generate raw data; the protein sequences were available through the NCBI database.
